# The Status of the Common, Cheap and Weak Drug

**Published:** 1914-08

**Authors:** 


**A Monthly Review of Medicine and Surgery**

EDITOR AND PUBLISHER, DR. A. L. BENEDICT, 228 Summer St., corner of Elmwood Ave., Buffalo, (Address for all communications. Please make personal and telephone calls before 1 P. M.)

Third, in age, on the western continent. Independent, but supports professional organization. News limited mainly to Western N. Y. and Penn.

Subscription $2.00 a year; $3.00 for two years in advance. Changes and errors in address or failure or duplication of delivery, should be reported immediately.

Yearly Volume 70. AUGUST, 1914 No. 1

### The Status of the Common, Cheap and Weak Drug

The title suggests a sweeping condemnation. Such adjectives are common in discussions of the ethical status of drugs. But, look through your tool case or instrument cabinet. Is there any logical connection between rarity, expense and size and strength and actual usefulness? As if to illustrate the point, we have just been interpreted to heal a lesion of the typewriter (neuter, not feminine) and have had to discard a powerful screw driver which was inefficient for a small, cheap one which effected a radical cure.

Let us take a few illustrations in the way of therapeutics, although these are somewhat objectionable as necessitating recourse to personal views. Quite a number of surgical cases die—mysteriously if the surgeon is too literally such and too certain of his own ability to have a medical review of the case —not from haemorrhage, sepsis, or shock, but simply because acidosis (an improper word), was overlooked and because large doses of ordinary sodium bicarbonate were not administered. It should be stated in qualification that acidosis is not a very common condition and that such treatment should not be employed as an unreasoning routine but only on actual demonstration of the condition. As an antacid in other conditions, this common domestic article is often far superior to acetates and citrates which become antacids only by decomposition into carbonates and whose conversion does not seem to be always prompt or dependable. Even in hyperchlorhydria—again with the proviso that the condition is actually demonstrated and not guessed at—the writer is gradually becoming sceptic as to the time-honored teaching of the superiority of the still alkalies, notably magnesium oxid or hydroxid, and depends, at least for the immediate relief of extreme degrees, almost entirely on sodium bicarbonate.

In various acute catarrhal conditions, an initial dose of catnip tea, which we are wont to despise as an old woman's remedy, acts promptly as a sudorific and, to use the old woman's expression with her remedy, "breaks up a cold." The same remedy, used by enema, seems also to be almost lifesaving in importance, in some cases of infantile convulsions.

One of the most agonizing cases that we have ever seen, was that of a little boy with acute proctitis of unexplained origin. Not only was it a condition of pain, but of about as high a degree of shock as is noted in medical cases. Both pain and danger were removed by a simple drug, absolutely inert so far as systematic action is concerned—purpetrol, introduced into the rectum.

For exophthalmic goitre, we have various methods from extirpation and removal of the cervical ganglia, through high frequency cataphoresis of silver salts, use of adrenalin, down, in the scale of dignity, to ointments and common sedatives. All things considered, we are inclined to rate a simple mixture of astringent chlorids given internally quite high in the scale of efficiency. The last method was discovered quite in accordance with the hope of the old empirics and the claim of modern quacks. Dr. L. B. Dorr was attending a case of offensive cancer, and was using a proprietary deodorant in a pan on the floor. The family had a large dog, afflicted with goitre. Coming in very thirsty, the dog lapped up the solution and condolences were extended in advance of the expected result. Some days later, the doctor observed that the family had bought another dog much resembling Curly, but free from goitre. He was assured that it was the same dog. Acting on this hint, we have, with some modification, adopted essentially the same method of treatment for goitre and even speak of it as the Curly method.

It is perfectly proper to raise questions of fact as to the adequacy of a dose, the activity of a particular preparation, or the pharmacologic effects of a drug. These questions have been raised with regard to cactus, even to the degree of attacking the ethical status of the drug itself. Yet, there are encountered many cardiac cases in which there is no indication either to increase the force of the heart's contraction or to alter the duration of its cycle or any part thereof. In the language of the old clinicians, what is needed is something to steady the heart. Now, save for certain hygienic and physical measures and for the dependence upon various anodynes and sedatives, for which there are obvious contraindications, the virtues ascribed to cactus fulfill the indications admirably. Whether empiric notions should be entirely set aside by pharmacologic experiment, is debatable. That cactus is a weak drug must be admitted even by those, among whom may be included many good clinicians, who do not consider it inert. But there can be no question of ethics attached to the use of this or any other drug in itself. The question is one of fact.

It is well to remember that most of the drugs of the highest standing, ethically and in the concession of powerful physiologic action, would fail, if the same conditions were applied to tests on them, as on drugs of slight physiologic action or those introduced as novelties and under circumstances which place them under the ethical ban. For example—and be it understood that we hold no brief either for the ethical or the therapeutic standing of the method—imagine that digitalis had been exploited, for cardiac affectations, as a novelty, by Dr. Friedmann.

It is, perhaps, an extreme view, but one toward which one leans more and more, as experience is acquired, that the possession of marked and easily demonstrated pharmacologic power by an active principle or a definite chemic action on tissues by a mineral drug, is rather a disqualification. If one limited such a statement to antipyretics, he would be perfectly safe. In extending it to morphine, atrophine, phosphorus, arsenic, bromides, etc., he would be voicing a heterodox view and we would not be understood as advocating such a view, except with many specifications.

Nevertheless, with regard to the danger of habit formation, with the realization that removal of pain, muscular spasm, wakefulness, etc., one has no more removed a disease than by controling the element of rise of temperature in a general fever, with the disquieting question as to the possibility of various degenerational lesions having arisen from previous "strong" medication, with the repeated observation that the apparently logical control of diseased conditions by powerful means, has produced the same ultimate effect as pushing a cork down into water, there results, either therapeutic nihilism or a certain degree of credulity in the value of very mild and harmless measures that modify disease only in an indirect way.

The latter tendency, carried to its extreme, again becomes eminently respectable and orthodox. If we substitute for a drug, a careful use of dietetic measures, as the administration of iron in meat rather than in a pill, or if we overcome excessive gastric secretion by bland food, or deficient secretion by dietetic stimulants, or if intestinal putrefaction by a radical change of pabulum or antagonizing bacteria or vaccines, our therapeutics is highly regarded. Especially is this true if we carry our therapeutics from the positive, through the zero of nihilism, to the negative phase of withdrawing certain substances, as in the salt-free diet, an excellent, eminently orthodox, and very indiscriminately and unwisely employed procedure.

We would state in conclusion, that in order to give emphasis, we have mentioned rather extreme cases and have purposely used the language of empirics, instead of attempting scientific explanations of some heterodox methods occasionally available. But the general proposition stands, that rarity, novelty, expense, and powerfulness of action do not, in general, render either a tool or a drug of superior value.

